# Estrogens revert neutrophil hyperplasia by inhibiting Hif1α-cMyb pathway in zebrafish myelodysplastic syndromes models

**DOI:** 10.1038/s41420-022-01121-2

**Published:** 2022-07-16

**Authors:** Xuexiao Li, Luping Wang, Xun Qin, Xiaohui Chen, Li Li, Zhibin Huang, Wenqing Zhang, Wei Liu

**Affiliations:** 1 Key Laboratory of Zebrafish Modeling and Drug Screening for Human Diseases of Guangdong Higher Education Institutes, Department of Developmental Biology, School of Basic Medical Sciences, Southern Medical University, Guangzhou, 510006 People’s Republic of China; 2 Department of Coloproctology, Zhujiang Hospital, Southern Medical University, Guangzhou, 510006 People’s Republic of China; 3 Division of Cell, Developmental and Integrative Biology, School of Medicine, South China University of Technology, Guangzhou, 510006 People’s Republic of China

**Keywords:** Drug development, Cancer prevention, Haematological cancer

## Abstract

Myelodysplastic syndromes (MDS) are characterized by daunting genetic heterogeneity and a high risk of leukemic transformation, which presents great challenges for clinical treatment. To identify new chemicals for MDS, we screened a panel of FDA-approved drugs and verified the neutrophil hyperplasia inhibiting role of 17β-estradiol (E2, a natural estrogen) in several zebrafish MDS models (*pu.1*^*G242D/G242D*^, *irf8*^*Δ57Δ/57*^ and *c-myb*^*hyper*^). However, the protective mechanism of estrogen in the development of hematological malignancies remains to be explored. Here, analyzing the role of E2 in the development of each hematopoietic lineage, we found that E2 exhibited a specific neutrophil inhibiting function. This neutrophil inhibitory function of E2 is attributed to its down-regulation of *c-myb*, which leads to accelerated apoptosis and decreased proliferation of neutrophils. We further showed that knockdown of *hif1α* could mimic the neutrophil inhibiting role of E2, and *hif1α* overexpression could reverse the protective function of E2. Collectively, our findings highlight the protective role of E2 on MDS by inhibiting *hif1α*-*c-myb* pathway, suggesting that E2 is a promising and effective drug for hematopoietic tumors associated with abnormal neutrophil hyperplasia.

## Introduction

Hematopoietic stem cell transplantation is a potential cure for refractory myelodysplastic syndromes (MDS) and acute myeloid leukemia (AML). Food and Drug Administration (FDA)-approved drugs are limited. Moreover, the existing drugs have inevitable adverse effects and can induce tolerance, owing to the disease’s heterogeneity and complex molecular underpinnings [1–3]. Compared with primary AML, AML developed from MDS is less sensitive to standard treatment and more prone to drug tolerance [3]. Therefore, identifying drugs targeting MDS and AML is extremely important. With rapid biotechnology developments, zebrafish have been widely applied as a model organism in drug screening for human diseases [4, 5]. Several key transcription factor defects have been reported to induce zebrafish MDS models with neutrophil hyperplasia, such as *pu.1*^*G242D/G242D*^, *irf8*^*Δ57Δ/57*^ and c-Myb hyperactive (*c-myb*^*hyper*^) [6–8].

In clinical settings, the hyperactivation of c-Myb and defects in Pu.1/Irf8 lead to various types of leukemia [9–12]. As previously reported in our study, *c-myb*^*hyper*^ zebrafish are characterized by hyperproliferation of neutrophils from embryonic to adult stages, and approximately 2% of *c-myb*^*hyper*^ fish in the adult stage develop acute myelocytic leukemia (AML), with a myelodysplastic syndrome (MDS)-like phenotype [8]. Because this MDS-like model appears the earliest and most obvious phenotype in the zebrafish embryo stage, we selected this pathological model to screen for new drugs for MDS/AML. In this study, we screened an FDA-approved drug library and found several estrogen drugs alleviated the phenotype of abnormally increased neutrophils in *c-myb*^*hyper*^ zebrafish.

Estrogens are a class of steroid compounds with a broad spectrum of biological activity. Three forms of natural estrogens have been found in vertebrates: estrone (E1), estradiol (E2) and estriol (E3). Among them, E2 has the strongest biological activity. According to clinical research, the incidence rate of hematological cancer is lower in women than in men [13], and the morbidity rate of chronic lymphocytic leukemia in men is almost twice that in women [14], thus suggesting that sex hormones may play important roles in preventing the development of hematological malignancies. After ovariectomy in rats, the decline in E2 levels can lead to hematopoietic dysfunction characterized by enhanced extramedullary hematopoiesis The administration of E2 to zebrafish in different hematopoietic periods displays opposite effects on hematopoietic stem and progenitor cells (HSPCs) [15]. Though E2 has been reported to improve hematological cancer and affect HSPCs, the underlying mechanisms remain largely undetermined.

In this study, we have provided clear evidence that E2 significantly decrease the abnormal neutrophil hyperplasia in zebrafish MDS models. Mechanistically, the neutrophil inhibitory function of E2 was partially on account of increased neutrophil apoptosis and decreased neutrophil proliferation. E2 inhibits *c-myb* expression dependent on down-regulation of *hif1α*. This research suggests that new strategies inhibiting *hif1α/c-myb* would be a promising treatment of hematopoietic tumors associated with neutrophil hyperplasia.

## Results

### Five estrogens were identified to inhibit neutrophil hyperplasia in zebrafish MDS models

**Fig. 1:**
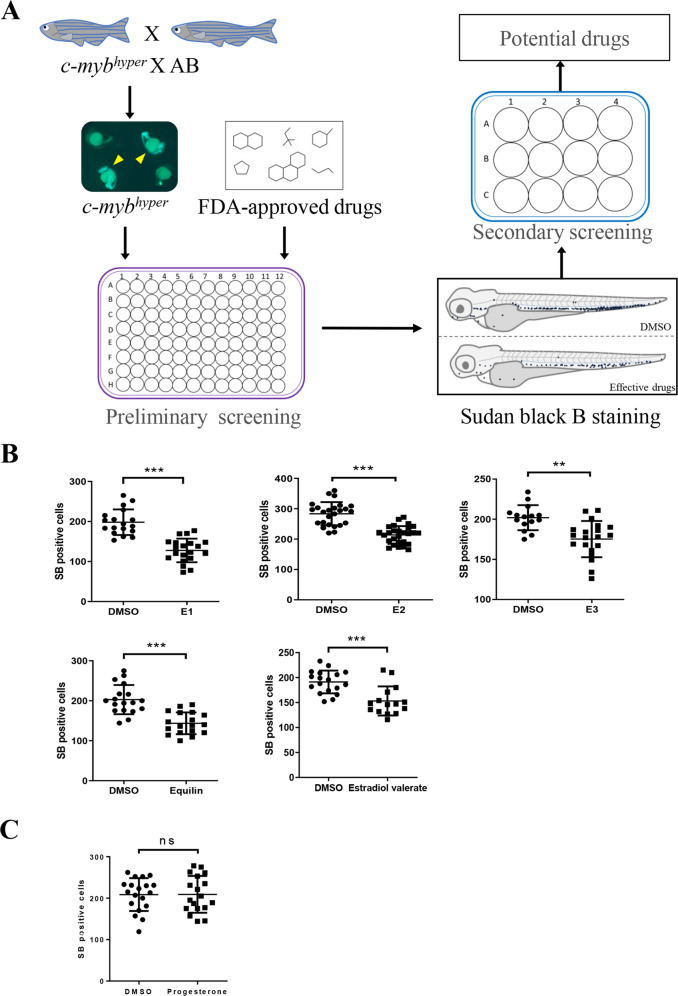
Estrogens decrease neutrophils in *c-myb*^*hyper*^ transgenic zebrafish. **A** Flowchart for drug screening in *c-myb*^*hyper*^ transgenic zebrafish. For preliminary screening, 96-well plates were used and each well was placed 5 embryos to detect potential effective drugs. For secondary screening, 12-well plates were used and each well was placed more embryos (*n* > 15) to further verify the potential effective drugs screened from the preliminary screening. **B** Natural estrogens decreased SB positive cells in the CHT region in *c-myb*^*hyper*^ transgenic zebrafish. Many pairs of zebrafish parents were selected for the drugs treatment, the embryos from each parent pair were randomly divided into two groups, one for a compound treated group and the other for its control (DMSO group). (*t*-test, ***p < 0.001, **p < 0.01. *n* > 15.) **C** Progesterone had no effect on SB positive cells in the CHT region in *c-myb*^*hyper*^ transgenic zebrafish. (*t*-test, ns, no significance. *n* > 15).

To obtain candidate targeted drugs able to alleviate the neutrophil hyperplasia in MDS, we subjected 1280 FDA-approved drugs to preliminary screening and secondary expanded screening in the *c-myb*^*hyper*^ zebrafish model (Fig. 1A). Neutrophils were counted through SB staining. Nine drugs alleviated the phenotype of increased SB^+^ cells in the caudal hematopoietic tissue (CHT) of *c-myb*^*hyper*^ zebrafish, five of which were estrogen compounds: E1, E2, E3, equilin and estradiol valerate (Fig. 1B and Supplementary Fig. S1A), and the remaining four drugs were chlorzoxazone, clopidogrel, nabumetone, and fenthion (data not shown). These results implied that estrogen drugs greatly decreased the number of neutrophils in *c-myb*^*hyper*^ zebrafish. However, progesterone, also a sex hormone drug, did not decrease the number of neutrophils (Fig. 1C and Supplementary Fig. S1A). These results suggested that neutrophil suppression was a specific effect of estrogen but not an extensive effect of sex hormones. Furthermore, to study whether estrogens inhibit neutrophil hyperplasia in other MDS models, we performed E2 treatment in the pathological models of *pu.1*^*G242D/G242D*^ and *irf8*^*Δ57Δ/57*^ zebrafish mutants, which had been previously established [6, 7]. In accordance with *c-myb*^*hyper*^ model, E2 alleviated the phenotype of increased SB^+^ cells in the two MDS models (Supplementary Fig. S1B, C), indicating that estrogens could revert the neutrophil hyperplasia of MDS in zebrafish.

### E2 decreases the number of neutrophils in *c-myb*^*hyper*^ zebrafish, mainly through accelerate apoptosis and decrease proliferation of neutrophils

**Fig. 2:**
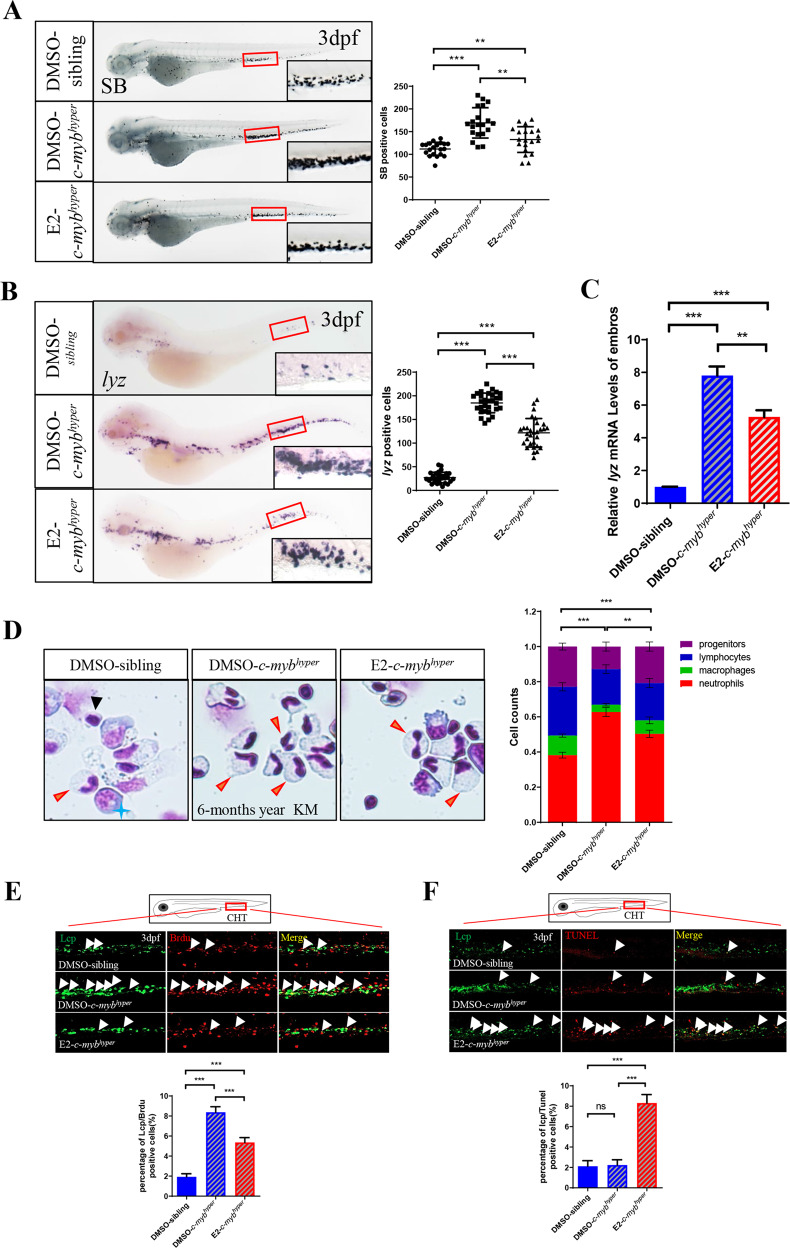
E2 decreases the number of neutrophils in *c-myb*^*hyper*^ zebrafish, mainly through inhibiting cell proliferation and promoting cell apoptosis. **A** E2 exposure decreased SB positive cells in the CHT region. (*t*-test, ***p < 0.001, **p < 0.01. *n* > 20). **B** E2 exposure decreased *lyz* in the CHT region, as determined by WISH. (*t*-test, ***p < 0.001, **p < 0.01. *n* > 20). **C** The qPCR quantification of the decrease in *lyz* expression with E2 (*t***-**test, mean ± SEM; ***p < 0.001, **p < 0.01. *n* ≥ 10). **D** May-Grunwald-Giemsa staining of whole KM blood cells in 6-month-old *c-myb*^*hyper*^ animals followed by four days of E2 treatment (*t*-test, ***p < 0.001. *n* = 12). Red arrowheads, blue asterisks, black arrowheads and yellow lightning indicate neutrophils, precursors, lymphocytes and macrophages, respectively. **E** Double staining of bromodeoxyuridine (BrdU)/Lcp indicated decreased neutrophil proliferation in *c****-****myb*^*hyper*^ zebrafish embryos treated with E2. (one-way ANOVA (LSD) ***p < 0.001, *n* = 12). **F** The TUNEL assays showed the effect of E2 on the apoptosis of myeloid lineage in zebrafish embryos (one-way ANOVA (LSD) ***p < 0.001, ns, no significance. *n* = 12).

To further analyze the effect of estrogen on hematopoietic development in *c-myb*^*hyper*^ zebrafish, we selected E2 (a natural estrogen and the most potent estrogen in humans) for subsequent experiments. First, we determined that the maximum tolerated concentration of E2 was 8 μM in zebrafish embryos (Supplementary Fig. S2A). Then, by treating *c-myb*^*hyper*^ zebrafish with different concentrations of E2 (8 μM, 6 μM, 4 μM and 2 μM) at 1 dpf for two days, we showed that E2 decreased neutrophils in *c-myb*^*hyper*^ zebrafish in a concentration-dependent manner (Supplementary Fig. S2B). In addition, the effects of E2 on neutrophils after different treatment times (one day and four days) were consistent with those after treatment for two days (Supplementary Fig. S2C, D). Therefore, we treated 1 dpf zebrafish with 8 μM E2 for two days as the following experimental conditions. We found that the number of neutrophils with SB staining (Fig. 2A) or *lyz/mpx* specific markers (Fig. 2B and Supplementary Fig. S3A) was significantly diminished in *c-myb*^*hyper*^ zebrafish but still greater than that in siblings. Consistently with these findings, the *lyz* mRNA expression in *c-myb*^*hyper*^ was also greatly decreased (Fig. 2C). Meanwhile, no clear changes were observed in *rag1*-labeled lymphocytes, *mfap4*-labeled macrophages and *βe1*-labeled erythrocytes in *c-myb*^*hyper*^ embryos after E2 treatment (Supplementary Fig. S3B–D). These data suggest that E2 has a neutrophil-specific hematopoietic effect on *c-myb*^*hyper*^ embryos. To further clarify whether E2 has an equal effect on neutrophils in adult zebrafish, we intraperitoneally injected E2 (2000 mg/kg) into adult male zebrafish (only males were used to eliminate the influence of endogenous estrogen in female zebrafish). The blood concentration of E2 after injection was estimated (Supplementary Fig. S3E), and the intraperitoneal injection of E2 was lasted for four days once daily. After E2 treatment, the proportion of neutrophils in kidney marrow (KM) in *c-myb*^*hyper*^ significantly declined (Fig. 2D). In summary, E2 significantly decreased the number of neutrophils in both *c-myb*^*hyper*^ embryos and adult fish.

To determine the cytological mechanisms through which E2 decreased the number of neutrophils in *c-myb*^*hyper*^, we detected the proliferation and apoptosis of neutrophils with BrdU and TUNEL staining, respectively. The results of BrdU and Lcp co-staining showed that the proportion of proliferative neutrophils (Lcp^*+*^BrdU^*+*^/Lcp^*+*^) in *c-myb*^*hyper*^ was far higher than that in siblings. And after E2 treatment, the proliferation of neutrophils in *c-myb*^*hyper*^ was significantly weakened (Fig. 2E), thus suggesting that E2 effectively decreased the hyperproliferation of neutrophils induced by abnormal activation of *c-myb*. Moreover, the TUNEL and Lcp co-staining showed no difference between the *c-myb*^*hyper*^ and sibling group in neutrophil apoptosis. While after E2 treatment, the neutrophil apoptosis in *c-myb*^*hyper*^ was significantly increased (Fig. 2F). In summary, E2 decreases the number of neutrophils in *c-myb*^*hyper*^ zebrafish, mainly through accelerated apoptosis and decreased proliferation of neutrophils.

### E2 decreases neutrophils in *c-myb*^*hyper*^ zebrafish independently of the classical estrogen receptor (ER) pathway

**Fig. 3:**
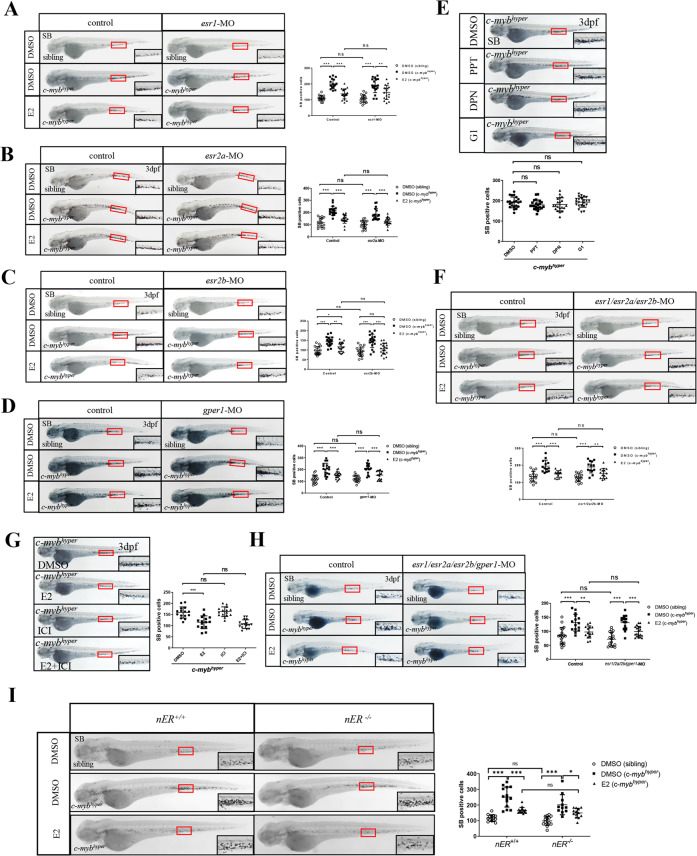
E2 decreases neutrophils in *c-myb*^*hyper*^ transgenic zebrafish embryos through ERs*-*independent mechanisms. **A**–**D** Knockdown of *esr1* (**A**)*, esr2a* (**B**)*, esr2b* (**C**) and *gper1* (**D**) did not alleviate E2-mediated inhibition of *c-myb*^*hyper*^ zebrafish neutrophils. (one-way ANOVA (LSD) ***p < 0.001, **p < 0.01, *p < 0.05. ns, no significance, *n* > 15). **E** PPT, DPN and G1 had no effect on *c-myb*^*hyper*^ zebrafish neutrophils. (one-way ANOVA (LSD), ns, no significance, *n* > 20). **F** Knockdown of *esr1, esr2a* and *esr2b* simultaneously did not alleviate E2-mediated inhibition of *c-myb*^*hyper*^ zebrafish neutrophils. (one-way ANOVA (LSD) ***p < 0.001, **p < 0.01, ns, no significance, *n* > 15). **G** ICI 182780 did not alleviate E2-mediated inhibition of *c-myb*^*hyper*^ zebrafish neutrophils. (one-way ANOVA (LSD) ***p < 0.001, ns, no significance, *n* > 15). **H** Knockdown of *esr1, esr2a esr2b* and *gper1* simultaneously did not alleviate E2-mediated inhibition of *c-myb*^*hyper*^ zebrafish neutrophils (one-way ANOVA (LSD) ***p < 0.001, **p < 0.01, ns, no significance, *n* > 15). **I** The triple *esr1*; *esr2a*; *esr2b* receptor mutant (esr^-/-^) was *p*roduced through mating to knock out all three classical nuclear receptors. SB staining showed that the E2-induced neutrophil decrease was also not alleviated in the triple mutant. (*t*-test, mean ± SEM; ***p < 0.001, *p < 0.05. ns, no significance. *n* > 10).

Estrogens are ligands that bind the ER and subsequently exert their classical biological effects. To explore whether E2 induced granulocytopenia in *c-myb*^*hyper*^ zebrafish is ER-dependent, we knocked down or activated the classical nuclear receptors (nER: *esr1*, *esr2a* and *esr2b*) and membrane receptor (mER: *gper1*) through MO micro-injection or reported agonist (ERα agonist PPT, ERβ agonist DPN and gpr30 agonist G1), respectively. The efficiency tests indicated that the MOs effectively blocked the expression of *esr1*, *esr2a* and *esr2b* (Supplementary Fig. S4A–C). The results showed that *esr1, esr2a*, *esr2b* and *gper1* knockdown alone did not block the E2-induced neutrophil decrease in *c-myb*^*hyper*^ (Fig. 3A–D), and activation of a single receptor also did not induce a neutrophil decrease in *c-myb*^*hyper*^ zebrafish (Fig. 3E). These data suggest that functional redundancy may exist among classical nuclear receptors and the membrane receptor, as previously reported [15, 16]. Therefore, we knocked down the nuclear receptors (nERs’ MO combined injection and nERs’ pan-antagonist ICI-182780 treatment) and all receptors (nERs+mERs’ MO combined injection), respectively. The results showed that the phenotype of neutrophil decrease in *c-myb*^*hyper*^ zebrafish embryos after E2 treatment was not reverted by joint ERs knockdown (Fig. 3F–H), which may due to the incompletely ER blocking by MO and antagonist. We further performed E2 treatment and SB staining on the *esr1*, *esr2a* and *esr2b* mutants reported previously [16, 17] and the *gper1* mutant obtained and validated through the CRISPR/CAS9 technique (Supplementary Fig. S4D–I). The E2-induced neutrophil decrease was not alleviated by single ER knockout (Supplementary Fig. S5A–D). Then, we generated a triple *esr1*; *esr2a*; *esr2b* receptor mutant (*nER*^*-/-*^), and tested the efficiency by detecting ER-responsive genes *vtg1/vtg3* [18]. The results showed that *vtg1* and *vtg3* expression significantly decreased in the triple mutant exposed to E2 (Supplementary Fig. S6A, B), but the E2-induced neutrophil decrease was also not alleviated (Fig. 3I). Collectively, these data imply that the neutrophil-reducing effect of E2 on *c-myb*^*hyper*^ zebrafish may not depend on the classical ERs.

### E2 alleviates neutrophil hyperplasia partially dependent on *c-myb* downregulation

**Fig. 4:**
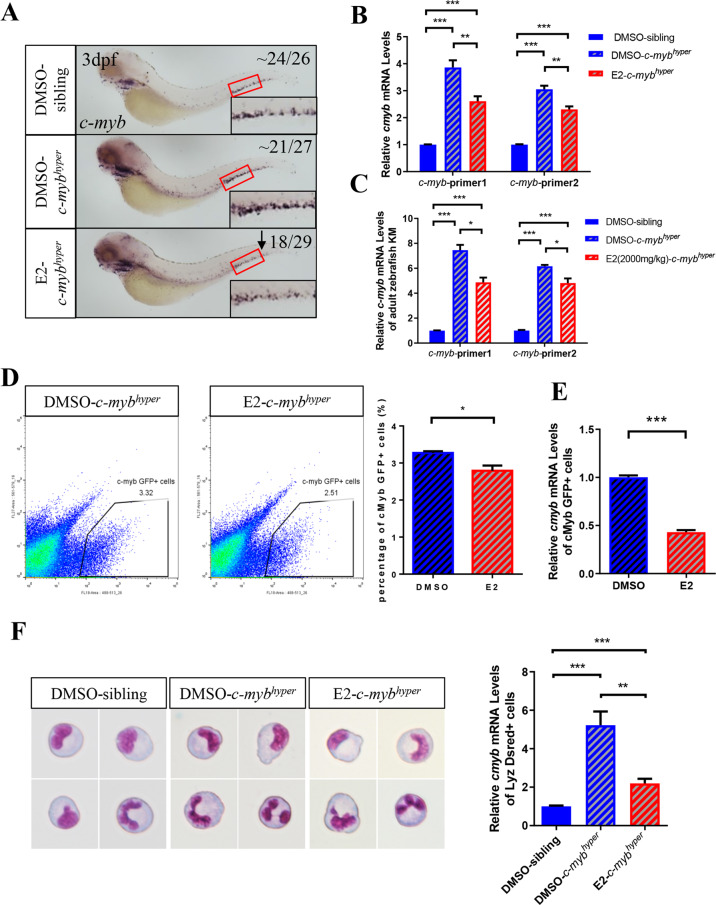
E2 decreases *c-myb* expression in *c-myb*^*hyper*^ transgenic zebrafish embryos. **A** E2 exposure decreased *c-myb* in the CHT, as determined by WISH. **B** qPCR quantification of the decreased *c-myb* expression by E2 (the *c-myb*^*hyper*^ fusion gene contained two parts, which comprised truncated *c-myb* from exon1 to exon10, followed by a near full-length *c-myb* from exon2 to exon 15). *c-myb*-primer1 and *c-myb*-primer2 were designed at repetitive and nonrepetitive sequences. (*t*-test, mean ± SEM. ***p < 0.001, **p < 0.01, *n* > 10). **C** qPCR quantification of decreased *c-myb* expression in *c-myb*^*hyper*^ adult zebrafish kidney after treatment with E2. (*t*-test, mean ± SEM. ^***^p < 0.001, ^*^p < 0.05, *n* > 10) (**D**) FACS analysis confirmed that E2 diminished *c-myb*:GFP^+^ cells. (*t*-test, mean ± SEM. ^*^p < 0.05, *n* > 10) (**E**) qPCR quantification of decreased *c-myb* expression in *c-myb*:GFP^+^ cells by E2. (*t*-test, mean ± SEM. ***p < 0.001, *n* > 10). **F** qPCR quantification of decreased *c-myb* expression in *lyz*:Dsred^+^ cells by E2. (*t*-test, mean ± SEM. ***p < 0.001, **p < 0.01, *n* > 10).

Previous studies have confirmed that *c-myb* is a key factor in neutrophil development [8, 19]. To study whether the inhibition of E2 in neutrophil development might depend on the expression of *c-myb*, we used WISH assays to detect *c-myb* expression after E2 treatment. After E2 treatment for 2 days, the numbers of *c-myb*^+^ cells in the CHT and AGM regions in *c-myb*^*hyper*^ zebrafish were significantly lower than those in the *c-myb*^*hyper*^-DMSO group, but still greater than those in the siblings (Fig. 4A). Consistently with these findings, real-time qPCR revealed that the expression of *c-myb* in *c-myb*^*hyper*^ whole embryos and adult KM also significantly decreased after E2 treatment (Fig. 4B, C). In addition, the FACS analysis showed that the proportion of *c-myb* GFP^+^ cells was lower in the *c-myb*^*hyper*^-E2 group than the *c-myb*^*hyper*^-DMSO group (Fig. 4D). However, the decrease in *c-myb*^+^ cells, based on WISH assays or FACS, was attributable to the decreased *c-myb* expression or the decrease in *c-myb*^*+*^ cells. To further determine the effect of E2 on the expression of *c-myb* per cell, we sorted *c-myb* GFP^+^ cells and detected the expression of *c-myb* by qPCR. The results revealed that the expression of *c-myb* in *c-myb* GFP^+^ cells of *c-myb*^*hyper*^ zebrafish greatly declined after E2 treatment (Fig. 4E). In addition, the expression of *c-myb* in sorted *lyz*^+^ neutrophils were also detected. In the *c-myb*^*hyper*^-E2 group, the expression of *c-myb* in neutrophils was significantly lower than that in the *c-myb*^*hyper*^-DMSO group but higher than that in the sibling-DMSO group (Fig. 4F). Therefore, we inferred that the alleviating effect of E2 on neutrophil hyperplasia was partially dependent on its down-regulation of *c-myb*.

### Hif1α participates in E2 induced down-regulation of *c-myb* and neutrophil hyperplasia

**Fig. 5:**
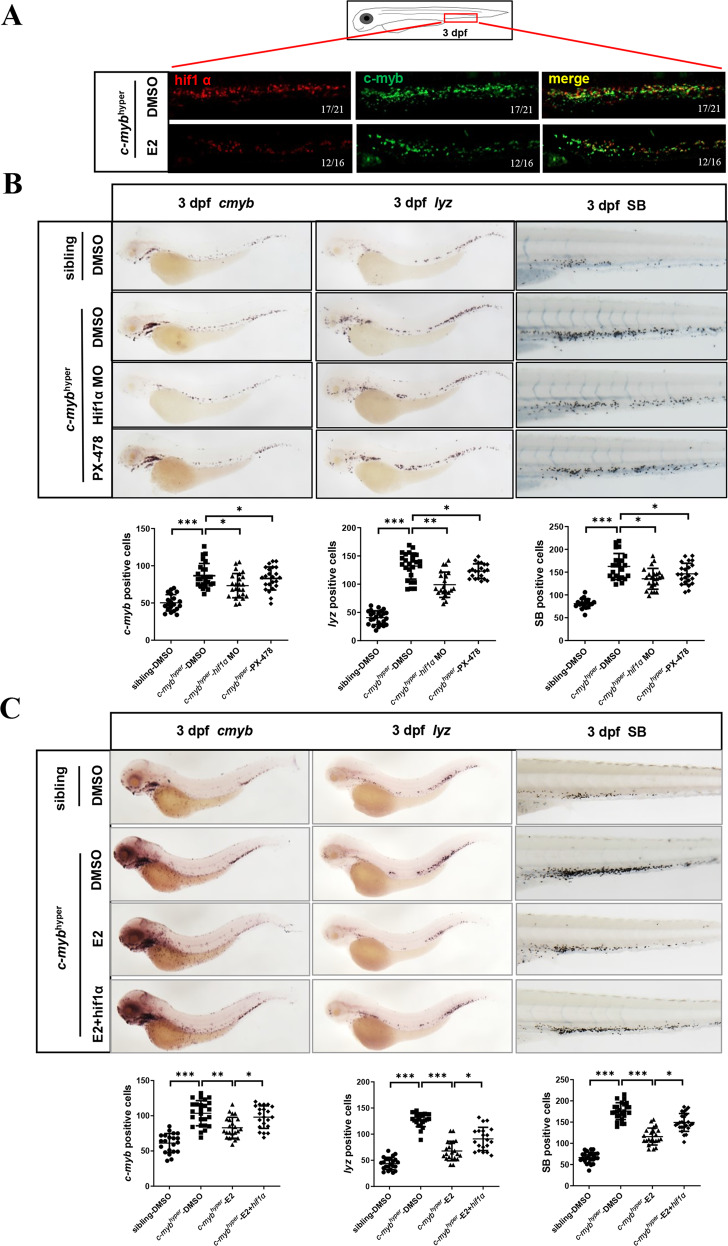
Hif1α participates in E2 induced down-regulation of *c-myb* and neutrophil hyperplasia. **A** Double staining of *hif1α* and *c-myb*-GFP with antibodies with or without E2 treatment. **B** Knockdown (MO) or inhibition (PX-478) of *hif1α* decreased *c-myb* and *lyz* expression by WISH as well as SB positive neutrophils in *c-myb*^*hyper*^ zebrafish. (*t*-test, ***p < 0.001, **p < 0.01, *p < 0.05, *n* > 20). **C** Overexpression of *hif1α* reversed E2 induced down-regulation of *c-myb* and *lyz* expression by WISH and neutrophil hyperplasia by SB staining. (*t*-test, ***p < 0.001, **p < 0.01, *p < 0.05, *n* > 20).

Interestingly, during the process of our drug screening, we found a compound PX-478 which was Hif1α inhibitor exhibited the neutrophil hyperplasia inhibiting role like E2 as well. It has been reported the Hif1/2 pathway functions upstream of Notch signaling in HSC formation (*runx1/c-myb*) [20]. Estrogen receptor-α directly regulates the Hif1 pathway associated with antiestrogen response in breast cancer [21]. We speculated that Hif1α may act as downstream of E2 and participate in the process of neutrophil hyperplasia. Immunofluorescence showed that the protein level of *hif1α* was decreased after E2 treatment in zebrafish (Fig. 5A). Embryos co-injected with *hif1aa* and *hif1ab* MOs (*hif1α* MO) exhibited a decrease in *c-myb* expression compared with negative control MO in *c-myb*^*hyper*^ zebrafish (Fig. 5B). We also found that the number of neutrophils with SB staining or *lyz* specific markers was significantly diminished in *c-myb*^*hyper*^ zebrafish (Fig. 5B). In accordance with genetic knockdown of *hif1α*, pharmacological inhibition with *hif1α* inhibitor PX-478 also exhibited the same results (Fig. 5B). To further investigate whether *hif1α* participates in E2 induced down-regulation of *c-myb* and neutrophil hyperplasia, we co-injected embryos with *hif1aa* and *hif1ab* (*hif1α*) overexpression plasmids. The results showed that *hif1α* overexpression could reverse E2 induced down-regulation of *c-myb* expression and neutrophil hyperplasia (Fig. 5C). Altogether, these observations suggest that Hif1α participates in E2 induced down-regulation of *c-myb* and neutrophil hyperplasia.

### E2 enhances neutrophil apoptosis through suppressing the expression of *hif1α* and *c-myb* under physiological conditions

**Fig. 6:**
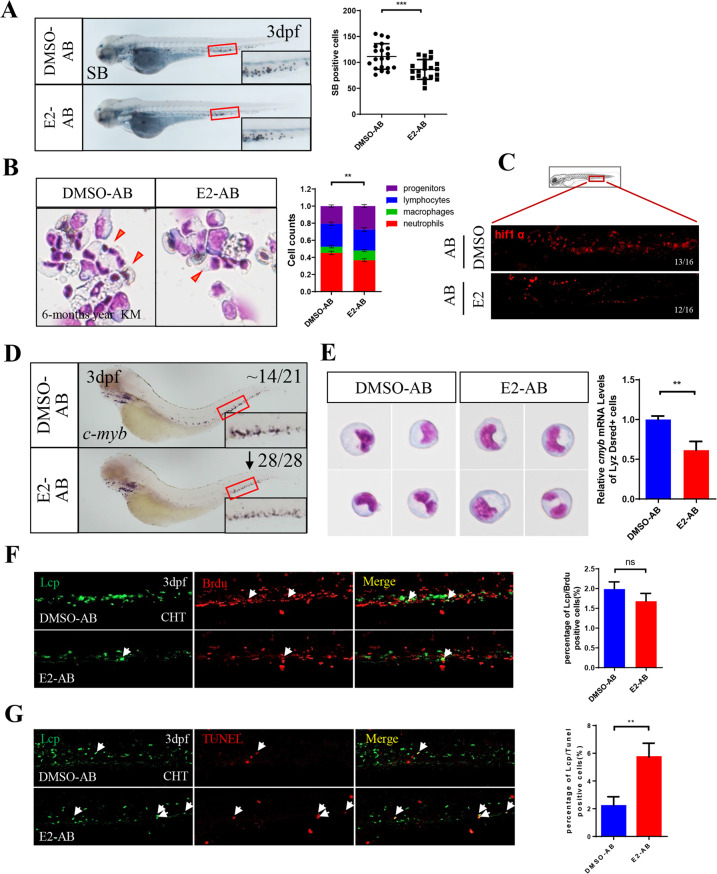
E2 enhances neutrophil apoptosis through suppressing the expression of *hif1α* and *c-myb* under physiological conditions. **A** E2 decreased neutrophils in AB zebrafish embryos. (*t*-test, ***p < 0.001, *n* > 20). **B** May–Grunwald–Giemsa staining of whole KM blood cells in 6-month-old AB zebrafish after 4 days of E2 treatment (*t*-test, ***p < 0.001, *n* = 12). Red arrowheads, blue asterisks, black arrowheads and yellow lightning indicates neutrophils, precursors, lymphocytes and macrophages, respectively. **C** Staining of *hif1α* with antibody with or without E2 treatment. **D** E2 exposure decreased *c-myb* in the CHT, as determined by WISH. **E** qPCR quantification of decreased *c-myb* expression in *lyz*:Dsred^+^ cells by E2 (*t*-test, mean ± SEM. ***p < 0.001, **p < 0.01, ^*^p < 0.05, *n* > 20). **F** Effect of E2 on neutrophil proliferation in AB zebrafish embryos. (one-way ANOVA (LSD). ns, no significance, *n* > 10). **G** E2 promotes the apoptosis of myeloid lineage cells in AB zebrafish embryos (one-way ANOVA (LSD) **p < 0.01. *n* > 10).

To clarify the effects of E2 on neutrophils under physiological conditions, we treated wild-type AB zebrafish with E2 for different times and detected the changes in neutrophils *via* SB staining. SB^+^ neutrophils were markedly decreased after treatment of E2 for 1–4 days (Fig. 6A and Supplementary Fig. S7A, B). Consistently, after E2 treatment for two days, *lyz-* and *mpx*-labeled neutrophils were also markedly decreased (Supplementary Fig. S7C–E), whereas other lineages remained unchanged (Supplementary Fig. S7F–H). In addition, the proportion of neutrophils in the AB KM was also decreased by E2 intraperitoneal injection once daily for four days (Fig. 6B), thus suggesting that, similarly to the results in *c-myb*^*hyper*^ zebrafish, E2 also specifically decreases the number of neutrophils under physiological conditions. Next, we detected the expression of *hif1α* and *c-myb* expression in AB after E2 treatment, IF showed that E2 significantly decreased *hif1α* expression (Fig. 6C). E2 treatment also significantly decreased the number of *c-myb*^+^ cells (Fig. 6D) and the overall expression of *c-myb* (Supplementary Fig. S7I, J) in AB zebrafish embryos. Furthermore, *lyz*^+^ neutrophils in AB zebrafish embryos treated with E2 were screened by FACS, and the expression of *c-myb* was confirmed be decreased, based on qPCR (Fig. 6E). These results demonstrated that E2 also diminished the number of neutrophils *via* suppressing the expression of *hif1α-c-myb* pathway under physiological conditions. Finally, we determined the cytological mechanism through which E2 decreased neutrophil counts by using BrdU and TUNEL staining. The results revealed that, in contrast to findings in *c-myb*^*hyper*^ zebrafish, the proliferation of neutrophils in AB zebrafish showed no significant changes after E2 treatment (Fig. 6F), whereas the apoptosis of neutrophils was significantly enhanced (Fig. 6G). These findings suggested that E2 can also regulate the *hif1α*-*c-myb* expression to promote neutrophil apoptosis, and lead to neutrophil decreased under physiological conditions.

## Discussion

**Fig. 7:**
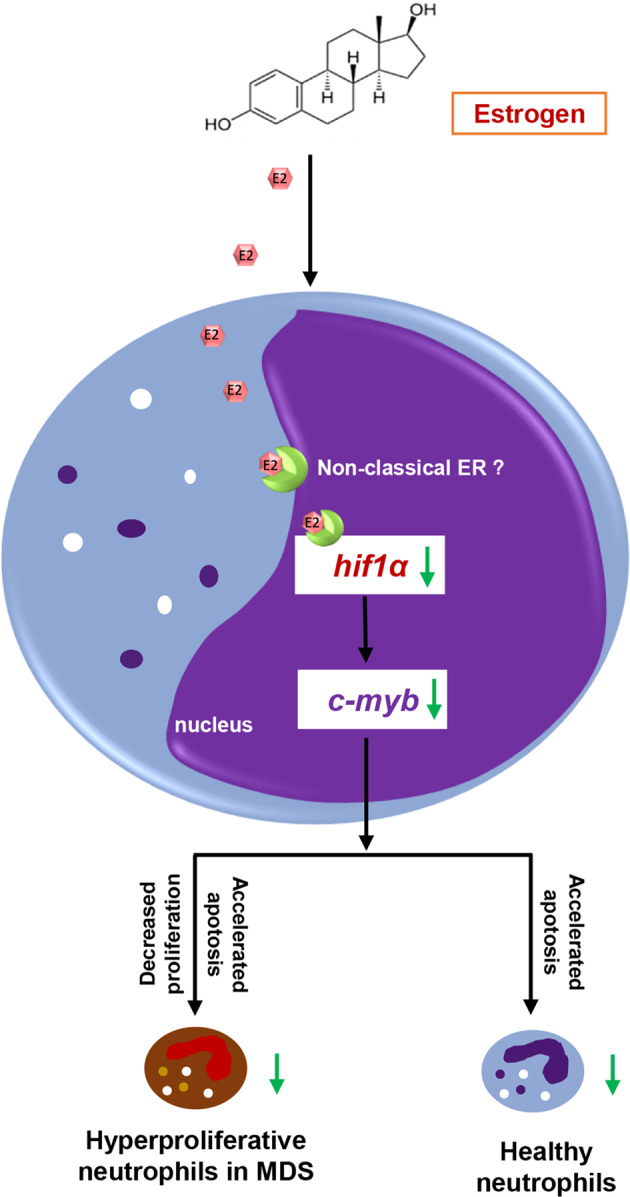
Schematic illustration of the neutrophil hyperplasia inhibiting role of E2 in zebrafish MDS E2 reverts neutrophil hyperplasia by regulating the proliferation and apoptosis of neutrophils through inhibition of the *hif1α-c-myb* pathway in zebrafish MDS models.

Existing chemotherapy drugs for MDS have ubiquitous adverse effects and drug resistance. Therefore, searching for alternative drugs is imperative. In this study, we found that estrogens significantly alleviated the blood phenotype of an abnormal increase in neutrophils in *c-myb*^*hyper*^ zebrafish embryos. Furthermore, E2 was found to regulate the proliferation and apoptosis of neutrophils in *c-myb*^*hyper*^ zebrafish through inhibiting *hif1α*-*c-myb* pathway, thereby decreasing the number of neutrophils (Fig. 7). This study demonstrated that estrogen may serve as a potential drug for hematologic diseases associated with abnormal neutrophil hyperplasia.

The regulatory effects of E2 in neutrophil development have been controversial in previous basic and clinical research. In vitro cell experiments have revealed that high dose of E2 inhibits the proliferation of bovine myeloid precursors, whereas low dose of E2 decreases the growth of neutrophil colonies [22]. Meanwhile, E2 promotes apoptosis and inhibits both chemotaxis and survival in bovine polymorphonuclear leukocytes in vitro [23], thereby indicating that estrogen restricts the number and function of leukocytes by affecting proliferation and apoptosis in vitro. In this study, we also demonstrated that neutrophil apoptosis is promoted in both wild-type and MDS zebrafish treated with E2. Notably, E2 only inhibits abnormal increased neutrophil proliferation in MDS zebrafish without affecting the normal neutrophil proliferation in wide-type zebrafish.

In addition, the role of estrogen in hematopoietic diseases has long been controversial. Traversa G et al. [24] have found that oral contraceptives are a potential risk factor for AML, but Poynter et al. [25] have analyzed the clinical data and found that the risk of postmenopausal AML declines in people who take oral contraceptives for more than five years. The reasons for these inconsistent research results may be associated with the region and age distribution of the population. Furthermore, AML is highly heterogeneous and has different clinical classifications and driver gene (including FLT3, CEBPA, U2AF1 and EZH2) abnormalities [26–28], which means different types of AML exhibit different responses to the same drug. In this study, we demonstrated E2 exerted a significant therapeutic effect in different zebrafish models of MDS (*c-myb*^*hyper*^*, pu.1*^*G242D/G242D*^ and *irf8*^*Δ57Δ/57*^), especially in the *c-myb*^*hyper*^ MDS model, thus demonstrating that E2 may be potential therapeutic drugs for hematological diseases associated with abnormal activation of *c-myb*. Though a previous study demonstrated *c-myb* expression was also increased in *pu.1*^*G242D/G242D*^ zebrafish [6], the underlying mechanisms of the protective role of E2 in different MDS models remain largely unknown. The future study would focus on whether E2 protects MDS *via* inhibiting *c-myb* expression in other zebrafish MDS models (such as *pu.1*^*G242D/G242D*^ and *irf8*^*Δ57Δ/57*^) with neutrophil hyperplasia as well.

In this study, we found the neutrophil-reducing effect of E2 may be independent of the known classical ERs. Such a classical ER-independent effect of E2 has also been reported in the development of mammalian breast tumors, the biological function and number of HSCs and the gonadal differentiation of zebrafish [16, 29–31], but the underlying mechanism remains unclear. The estrogen-related receptors (ESRR, including ERRα, ERRβ, and ERRγ) pathway is a non-classical estrogen signal transduction pathway, which share similar DNA binding sequences and not identical ligands with ERs [32, 33]. In this study, we demonstrated that E2 decreased *c-myb* expression and the number of neutrophils by regulating the level of *hif1α*. Therefore, whether ESRRs and other unknown ERs might be involved in the inhibition of E2 on the *hif1α* remains to be further explored.

## Materials and methods

### Zebrafish husbandry

All experiments involving zebrafish were performed in accordance with the guidelines of the Institutional Animal Care and Use Committee of Southern Medical University. Zebrafish (3–5 days) were maintained as described previously [34]. AB strain zebrafish was applied as wild-type animals. The following strains were used: *c-myb*^*hyper*^, *Tg(lyz:DsRed)*, *pu.1*^*G242D/G242D*^ and *irf8*^*Δ57Δ/57*^. *esr1* [17], *esr2a* [16] and *esr2b* [16] mutant zebrafish were received from the Faculty of Health Sciences, University of Macau, and evaluated by genotyping as previously described. The genotyping primers (HRM) for *esr1*, *esr2a* and *esr2b* were listed in Supplementary Table 1.

### Generation of *gper1*-mutant line and validation

CRISPR/Cas9 was utilized to create *gper1* mutants. The gRNA (*gper1*: 5ʹ- GGATGGAGGCCATCCAGATG-3ʹ) was co-injected with Cas9 protein (NEB, MA, United States; M0646M) into one-cell stage embryos, the gRNA was synthesized as described [35]. The raising and screening of mutants were performed as previously described [35]. Identified F1 and progeny were used for experiments. *gper1*^*+/+*^ and *gper1*^*−/−*^ embryos were generated and genotyped from heterozygous intercrosses. The genotyping primers (HRM) for *gper1* were listed in Supplementary Table 1.

### Chemical treatments

The FDA approved 1280 drugs for our initial screening were listed in supplementary drugs information. The *c-myb*^*hype*r^ and/or sibling embryos were identified through fluorescence under a Zeiss microscope at 24 hpf. Drugs dissolved in DMSO (stock concentration: 10 mM) were added in egg water (work concentration: 100 uM) to treat embryos from 24 hpf until 3 dpf or 5 dpf in multi-well plates. All drugs were absorbed by zebrafish embryos through immersion. For two days of drug treatment (drugs were added at 24 hpf, and the effect was detected at 3 dpf), we did not replace the water. For four days of drug treatment (drugs were added at 24 hpf, and the effect was detected at 5 dpf), the water was replaced and the drug was reintroduced at the 3 dpf. Adult fish were intraperitoneally injected with E2 (2000 mg/kg) once daily for four days. The concentration for intraperitoneal injection was the maximum acceptable concentration (2 mM) in phosphate buffered saline. The following compounds were used: DMSO (Sigma-Aldrich, D2650); 17β-estradiol (E2:8 µM, Selleck, S1709); *2,3-bis*(4-hydroxyphenyl) propionitrile (DPN:50 µM, glpbio, GC17688); propyl pyrazole triol (PPT: 50 µM, glpbio, GC14370); fulvestrant (ICI-182,780: 15 µM, glpbio, GC18000), G1 (1 µM, MCE, HY-107216), E2 ELISA kit (Laier Biotechnology, LE-Y256) and PX-478 (20 µM, MCE, HY-10231) or compounds from the compound library (TargetMol).

### Sudan Black staining

Fixed embryos subjected to chemical treatments as detailed above were incubated in Sudan Black B (SB) (Sigma-Aldrich, St. Louis, MO, USA; 199664) solution and washed as previously described [36].

### Whole mount in situ hybridization (WISH)

The *c-myb*^*hyper*^ and sibling embryos were treated for two days or four days, fixed at 3 dpf or 5 dpf in 4% paraformaldehyde, and processed with antisense digoxigenin-labeled RNA probes as previously described [37]. Images were captured with a Zeiss Axio Zoom.V16, Zeiss Axio Imager.C1.

### May–Grünwald–Giemsa staining of kidney marrow cells

The zebrafish was placed on ice for anesthesia, and the kidney was removed and placed in PBS containing 5% FBS. The removed kidneys were gently pipetted to disperse the hematopoietic cells accumulated in the renal tubules. The blown cells were filtered through a filter with 40-µm pore size, and the cell suspension was added to the cytospin onto slides, centrifuged at 400 rpm for 3 min. The slides were then airdried and subjected to May–Grünwald–Giemsa (Sigma-Aldrich, May-Grünwald solution, 63950 and Giemsa solution, 32884) staining according to the standard protocol.

### Fluorescence activated cell sorting (FACS) analysis

The *c-myb*^*hyper*^ and/or sibling embryos after E2 treatment for two days were collected at 3 dpf, collected with a filter with 100-µm pore size, washed and resuspended in 0.9× phosphate buffered saline plus 5% FBS, digested, and then passed through a filter with 40-µm pore size. Flow cytometry analysis and sorting were based on forward scatter and side scatter with a flow cytometer (Beckman Coulter MoFlo XDP).

### Total RNA extraction and quantitative RT-PCR

Total RNA was extracted with TRIzol reagent according to the manufacturer’s instructions. cDNA was transcribed with M-MLV Reverse Transcriptase (Promega, M1701). All assays were performed in duplicate or triplicate. The qPCR reactions were performed with a LightCycler 96 PCR instrument (Roche). The relative gene expression was calculated with the 2^−ΔΔCt^ method, with normalization to the level of elongation factor 1α (*ef1α*). Primers were designed by Primer 5 and the primer sequences were listed in Supplementary Table 1.

### Bromodeoxyuridine (BrdU) labeling

BrdU labeling was performed as described previously [36]. Embryos were incubated with 10 mM BrdU (Sigma-Aldrich; B5002) for 2 h, and then stained with mouse anti-BrdU (Roche, 11170376001, 1:50) and rabbit anti-dsRed (Clontech, 632496, 1:100), followed by Alexa Fluor anti-mouse 488 (Invitrogen, A21202, 1:200) and anti-rabbit 555(Invitrogen, A31572, 1:200) for fluorescence visualization. Images were captured with Zeiss LSM800 confocal microscope system.

### Terminal deoxynucleotidyl transferase dUTP nick end labeling (TUNEL) assay

TUNEL assays were performed with an In Situ Cell Death Detection Kit (Roche, 12156792910), and this was followed by incubation with rabbit anti-Lcp (GeneTex, GTX124420, 1:200) and Alexa Fluor anti-rabbit 555 (Invitrogen, A31572, 1:200). Images were captured with Zeiss LSM800 confocal microscope system.

### Double fluorescence immunohistochemistry staining

Immunohistochemistry was performed essentially as described previously [38]. To examine the co-staining of green fluorescent protein (GFP) and Hif1α, the embryos were first stained with goat anti-GFP (Abcam, ab6658, 1:400), and rabbit anti-Hif1α antibody (Novus Biologicals, NB100-134, 1:250) and were subsequently visualized by AlexaFluor-488 donkey anti-goat (Invitrogen, A32814, 1:200) for GFP and AlexaFluor-555 donkey anti-rabbit (Invitrogen, A31572, 1:200) for Hif1α.

### Morpholino (MO) injections

MOs (Gene Tools) were designed according to references [39, 40] and injected into one cell stage embryos. The MO oligo was diluted to a concentration of 1 mM and injected into one-cell-stage embryos. This was followed by E2 treatment as detailed above. The MO oligo sequences were listed in Supplementary Table 2.

### Generation of the *hif1aa/ab* overexpression construct

For the *pTAL-ef1a-hif1aa/ab-dsRed* construct, *hif1aa/ab* cDNA containing the coding region but not the stop codon was cloned into the pTAL vector under the control of the *ef1a* promoter, with *dsRed* fused after *hif1aa/ab* cDNA. For overexpression of hif1aa/ab-dsRed, 50 ng/μl of DNA construct and 40 ng/μl transposase mRNA were co-injected into one-cell-stage embryos.

### Statistical analysis

Data were analyzed in SPSS software (version 15.0). Student’s *t*-tests, Fisher’s exact test and one-way analysis of variance (ANOVA) with Tukey’s adjustment were used to compare differences between groups. *P* < 0.05 was considered to indicate significance. Data are expressed as mean ± standard deviation (SD) unless otherwise indicated.

## Supplementary information

SUPPLEMENTARY FIGURES AND TABLES

SUPPLEMENTARY DRUGS INFORMATION

## Data Availability

All data generated or analyzed during this study are included in this published article and its supplementary information files. The datasets used and analyzed during the current study are available from the corresponding author on reasonable request.
