# Supplementary material for: Estrogens revert neutrophil hyperplasia by inhibiting Hif1α-cMyb pathway in zebrafish myelodysplastic syndromes models
Source: Cell Death Discov. 2022 Jul 16;8:323. doi: 10.1038/s41420-022-01121-2 (PMC9288432; doi:10.1038/s41420-022-01121-2)
Supplement: Supplementary file 1 — SUPPLEMENTARY FIGURES AND TABLES [file 41420_2022_1121_MOESM1_ESM.docx]

**SUPPLEMENTARY MATERIAL**

**Estrogens revert neutrophil hyperplasia by inhibiting Hif1α-cMyb pathway in zebrafish myelodysplastic syndromes models**

Xuexiao Li,^1,^^2^* Luping Wang,^3^* Xun Qin,^1^ Xiaohui Chen,^3^ Li Li,^3^ Zhibin Huang,^3^ Wenqing Zhang,^1,3^**^#^** and Wei Liu^3^**^#^**

^1^Key Laboratory of Zebrafish Modeling and Drug Screening for Human Diseases of Guangdong Higher Education Institutes, Department of Developmental Biology, School of Basic Medical Sciences, Southern Medical University, Guangzhou 510006, People’s Republic of China.

^2^Department of Coloproctology, Zhujiang Hospital, Southern Medical University, Guangzhou 510006, People’s Republic of China.

^3^Division of Cell, Developmental and Integrative Biology, School of Medicine, South China University of Technology, Guangzhou 510006, People’s Republic of China.

*XL and LW contributed equally as co-first authors.

**^#^ Corresponding authors:**

W.L: Laboratory of Developmental Biology and Regenerative Medicine, School of Medicine, South China University of Technology, Guangzhou 510006, China. Tel/Fax: 86-20-39380971; Email: <liuwei7@scut.edu.cn.> ORCID: <https://orcid.org/0000-0003-3660-5514>.

W.Q.Z: Department of Developmental Biology, School of Basic Medical Sciences, Southern Medical University; Laboratory of Developmental Biology and Regenerative Medicine, School of Medicine, South China University of Technology, Guangzhou 510006, China. Tel/Fax: 86-20-39380971; Email: <mczhangwq@scut.edu.cn.> ORCID: <https://orcid.org/0000-0002-3636-7133>.

**Supplementary Fig. S1. SB staining of *c-myb^hyper^* transgenic zebrafish after exposure to natural estrogens and exploration of suitable concentrations of E2 for treatment.** (**A**) SB staining of *c-myb^hyper^* transgenic zebrafish after exposure to natural estrogens and progesterone. (**B**) E2 decreased neutrophils in both widetype and *pu.1^G242D/G242D^* mutant zebrafish by SB stanning. (*t*-test, mean±SEM. ^***^*p*<0.001, *n*>15). (**C**) E2 decreased neutrophils in both widetype and *irf8^Δ57Δ/57^* mutant zebrafish by SB stanning. (*t*-test, mean±SEM. ^***^*p*<0.001, ^**^*p*<0.01, *n*>15).

**Supplementary Fig. S2.** (**A**) Left: Survival curve of *c-myb^hyper^* zebrafish embryos with E2 treatment. Right: Abnormality rate of *c-myb^hyper^* zebrafish embryos with E2 treatment. (**B**) Analysis of SB positive cells in the CHT region in *c-myb^hyper^* zebrafish embryos after E2 exposure at different concentrations (one-way ANOVA (LSD) ^***^*p*<0.001, ^**^*p*<0.01, ^*^*p*<0.05. ns, no significance, *n*>20). (**C**) E2 exposure for 24 h decreased SB positive cells in the CHT region (*t*-test, ^***^*p*<0.001, ^**^*p*<0.01, *n*>15). (**D**) E2 exposure for 4 d decreased SB positive cells in the CHT region (*t*-test, ^***^*p*<0.001, *n*>15).

**Supplementary Fig. S3. E2 decreases neutrophils in *c-myb^hyper^* zebrafish embryos without affecting macrophage, erythrocyte and lymphocyte markers.** (**A**) E2 exposure decreased*mpx* in the CHT region, as determined by WISH. (*t*-test, ^***^*p*<0.001, *n*>20). (**B**) WISH of lymphocyte markers in *c-myb^hyper^* zebrafish after E2 exposure. The area of *rag1^+^* signals was determined by imageJ. (*t*-test, ^***^*p*<0.001, ns, no significance, *n*>20) (**C**) WISH of *mfap4* in *c-myb^hyper^* zebrafish after E2 exposure (*t*-test, mean±SD; ^***^*p*<0.001; ns, no significance, *n*>20). (**D**) WISH of *βe1* in *c-myb^hyper^* zebrafish after E2 exposure. Two groups were classified by the signal intensity. Gray represents the number of embryos with less expression of *βe1*. Dark gray represents the number of embryos with more expression of *βe1*. The numbers in the corner of the image indicated the number of embryos showing more signals (left) and the whole embryos (right). (Fisher’s exact test, *n*>15). (**E**) E2 (2000 mg/kg) was intraperitoneally injected into adult male zebrafish (only males were used to eliminate the influence of endogenous estrogen in female zebrafish), the blood concentration of E2 was detected after injection by Fish E2 ELISA kit (Laier Biotechnology, LE-Y256) at several time points (0.5h, 1.5h, 6h, 12h). (*n*>10).

**Supplementary Fig. S4. Schematic diagram of the nERs MOs target locus and the efficiency test of the three MOs in** ***c-myb^hyper^* zebrafish** **embryos, and SB** **staining of *c-myb^hyper^* transgenic zebrafish embryos after ER-modulator treatment.** (**A**) Schematic diagram of the *esr1* morpholino target locus and the efficiency test of the *esr1* morpholino in *c-myb^hyper^* zebrafish. L. Schematic diagram of the *esr1* gene, showing translated and untranslated exons (shaded and unshaded boxes, respectively; I–IX), approximate locations of the oligonucleotide MO (red bar) and primers used for q-PCR (F1 and R1). R. qPCR quantification of decreased *esr1* mRNA expression in *c-myb^hyper^* zebrafish embryos after *esr1* morpholino injection. (*t*-test, ^***^*p*<0.001, *n*>15). The left red bar represents *esr2a* or *2b* morpholino injection, and the right red bar represents *esr1* morpholino injection (*t*-test, mean±SEM. ^***^*p*<0.001，*n*>15). DBD, DNA-binding domain; LBD, ligand binding domain. (**B**) Schematic diagram of the *esr2a* morpholino target locus and the efficiency test of *esr2a* morpholino in *c-myb^hyper^* zebrafish. L. Schematic diagram of the *esr2a* gene. R. qPCR quantification of decreased *esr2a* mRNA expression in *c-myb^hyper^* zebrafish embryos after *esr2a* morpholino injection. The left red bar represents *esr1* or *2b* morpholino injection, and the right red bar represents *esr2a* morpholino injection (*t*-test, ** *p*<0.01, *n*>15). (**C**) Schematic diagram of the *esr2b* morpholino target locus and the efficiency test of *esr2b* morpholino in *c-myb^hyper^* zebrafish. L. Schematic diagram of the *esr2b* gene. R. qPCR quantification of decreased *esr2b* mRNA expression in *c-myb^hyper^* zebrafish embryos after *esr2b* morpholino injection. (*t*-test, ^***^*p*<0.001, *n*>15). The left red bar represents *esr1* or *2a* morpholino injection, and the right red bar represents *esr2b* morpholino injection (*t*-test, mean±SEM. ^***^*p*<0.001. *n*>15). (**D**-**F**) Targeted disruption of the zebrafish *esr1/esr2a/esr2b* gene. Sequencing results of the *esr* gene from wild-type (*esr*^+/+^) and homozygous (*esr*^−/−^) zebrafish.(**G**) Site-specific targeting for CRISPR/Cas9 cleavage within exon Ⅲ of the zebrafish *gper1* gene. Alignment of nucleotide sequences from wild-type and mutant *gper1* alleles in *gper1* (-7, +5) zebrafish line. Dashes in DNA sequences are the nucleotides deleted during repair of CRISPR/Cas9-induced double-strand breaks. PAM: protospacer adjacent motif. (**H**) DNA sequencing identified a deletion of 7 bases and insert of 5 bases in the third exon of *gper1*. (**I**) Real-time PCR analysis shows almost undetectable *gper1* mRNA in *gper1* mutants at 3 dpf. (t-test, ^***^*p*<0.001, *n*>15).

**Supplementary Fig. S5. Modulation of ERs does not alleviate E2-mediated inhibition of zebrafish neutrophils.** (**A**) SB staining showing that knockout of *esr1* did not alleviate E2-mediated inhibition of wild-type zebrafish neutrophils. (*t*-test, mean±SEM. ^***^*p*<0.001, ^**^*p*<0.01, ^*^*p*<0.05. ns, no significance, *n*>10). (**B**) SB staining showing that knockout of *esr2a* did not alleviate E2-mediated inhibition of wild-type zebrafish neutrophils. (*t*-test, mean±SEM. ^***^*p*<0.001. ns, no significance, *n*>12). (**C**) SB staining showing that knockout of *esr1* did not alleviate E2-mediated inhibition of wild-type zebrafish neutrophils. (*t*-test, mean±SEM. ^***^*p*<0.001, ^**^*p*<0.01. ns, no significance, *n*>12). (**D**) SB staining showing that knockout of *gper1* did not alleviate E2-mediated inhibition of wild-type zebrafish neutrophils. (*t*-test, mean±SEM. ^***^*p*<0.001. ns, no significance, *n*>12).

**Supplementary Fig. S6. *vtg1* and *vtg3* expression were significantly decreased in the *esr1/esr2a/esr2b^-/-^* triple mutant (nER^-/-^) exposed to E2.** (**A** and **B**) qPCR quantification of decreased *vtg1 and vtg3* expression in triple mutant (nER^-/-^) exposed to E2 compared with the widetype (nER^+/+^). (*t*-test, mean±SEM. ^***^*p*<0.001, ^*^*p*<0.05. ns, no significance, *n*>12).

**Supplementary Fig. S7. Effects of E2 on blood phenotype and *c-myb* expression on wild-type AB zebrafish.** (**A**) E2 exposure for 24 h decreased SB positive cells in the CHT region in AB zebrafish. (*t*-test, mean±SEM. ^***^*p*<0.001, *n*>20). (**B**) E2 exposure for 4 days decreased SB positive cells in the CHT region in AB zebrafish. (*t*-test, mean±SEM. ^***^*p*<0.001, *n*>20). (**C**) E2 exposure decreased*lyz* in the CHT region in AB zebrafish, as determined by WISH. (*t*-test, mean±SEM. ^***^*p*<0.001, *n*>15). (**D**) E2 exposure decreased *mpx* in the CHT region in AB zebrafish, as determined by WISH (*t*-test, ^***^*p*<0.001, *n*>15). (**E**) qPCR quantification of decreased *lyz* expression in AB zebrafish by E2 (*t*-test, mean±SEM; ^***^*p*<0.001, *n*>15). (**F**) WISH of lymphocyte markers in AB zebrafish after E2 exposure (Fisher’s exact test, *n*=20). (**G**) WISH of erythrocyte markers in AB zebrafish after E2 exposure (Fisher’s exact test, *n*>15). (**H**) WISH of macrophage markers in AB zebrafish after E2 exposure (*t*-test, ns, no significance, *n*>20). (**I**) The qPCR quantification of decreased *c-myb* expression in AB zebrafish treated with E2. (*t*-test, mean±SEM. ^**^*p*<0.01. *n*>10). (**J**) The qPCR quantification of decreased *c-myb* expression in AB adult zebrafish kidneys after treatment with E2 (*t*-test, mean±SEM. ^*^*p*<0.05, *n*>10).


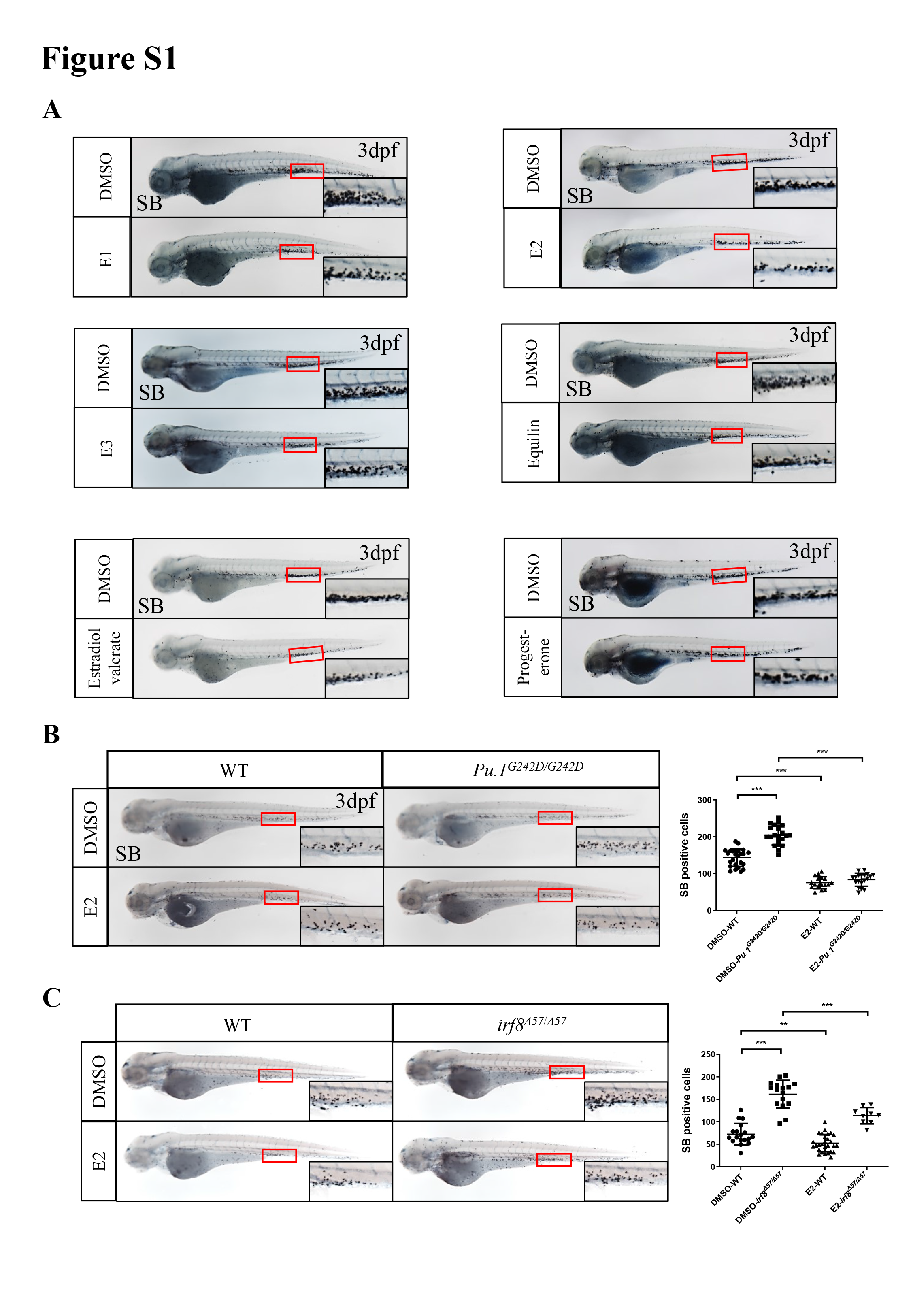


**
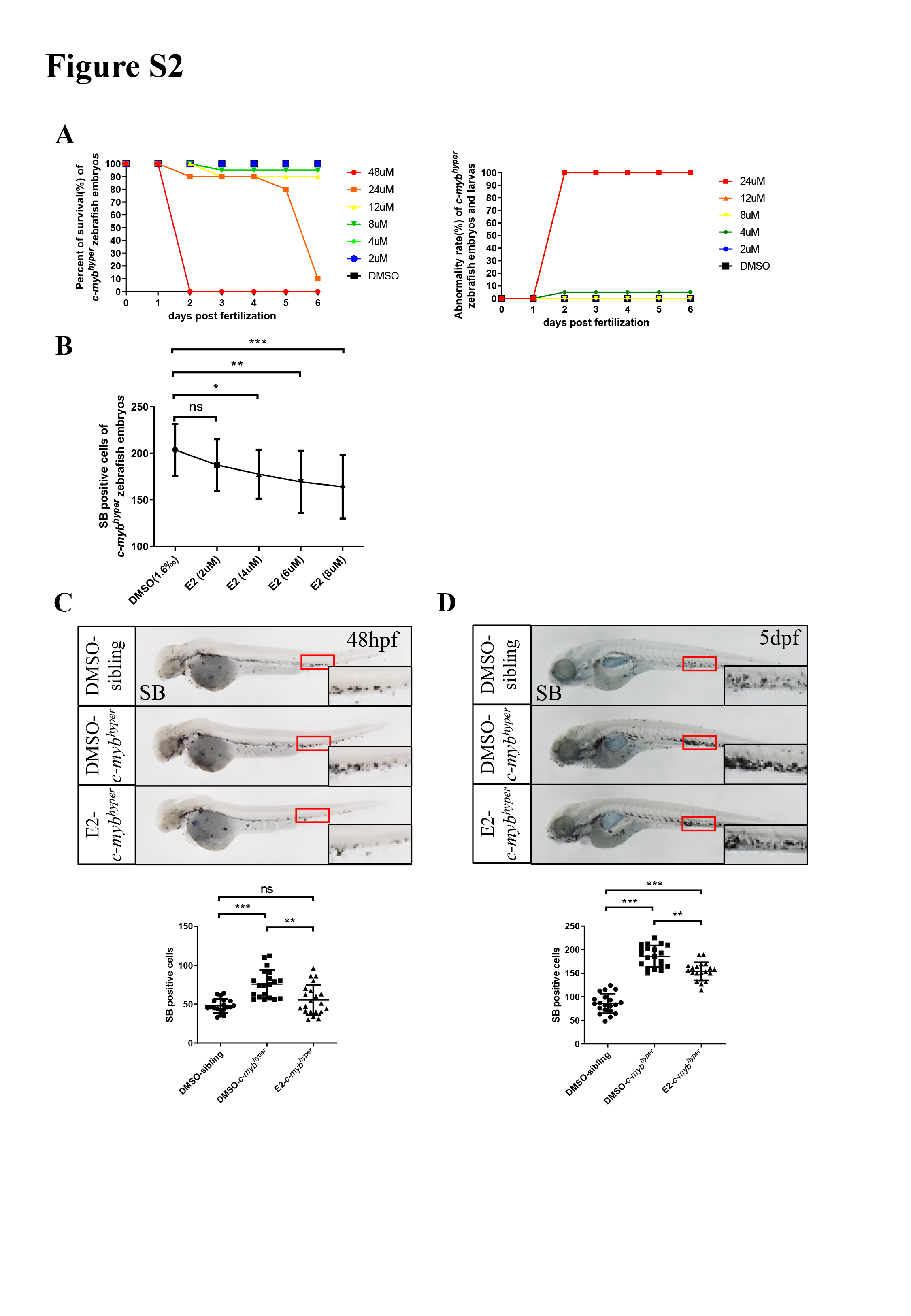
**

**
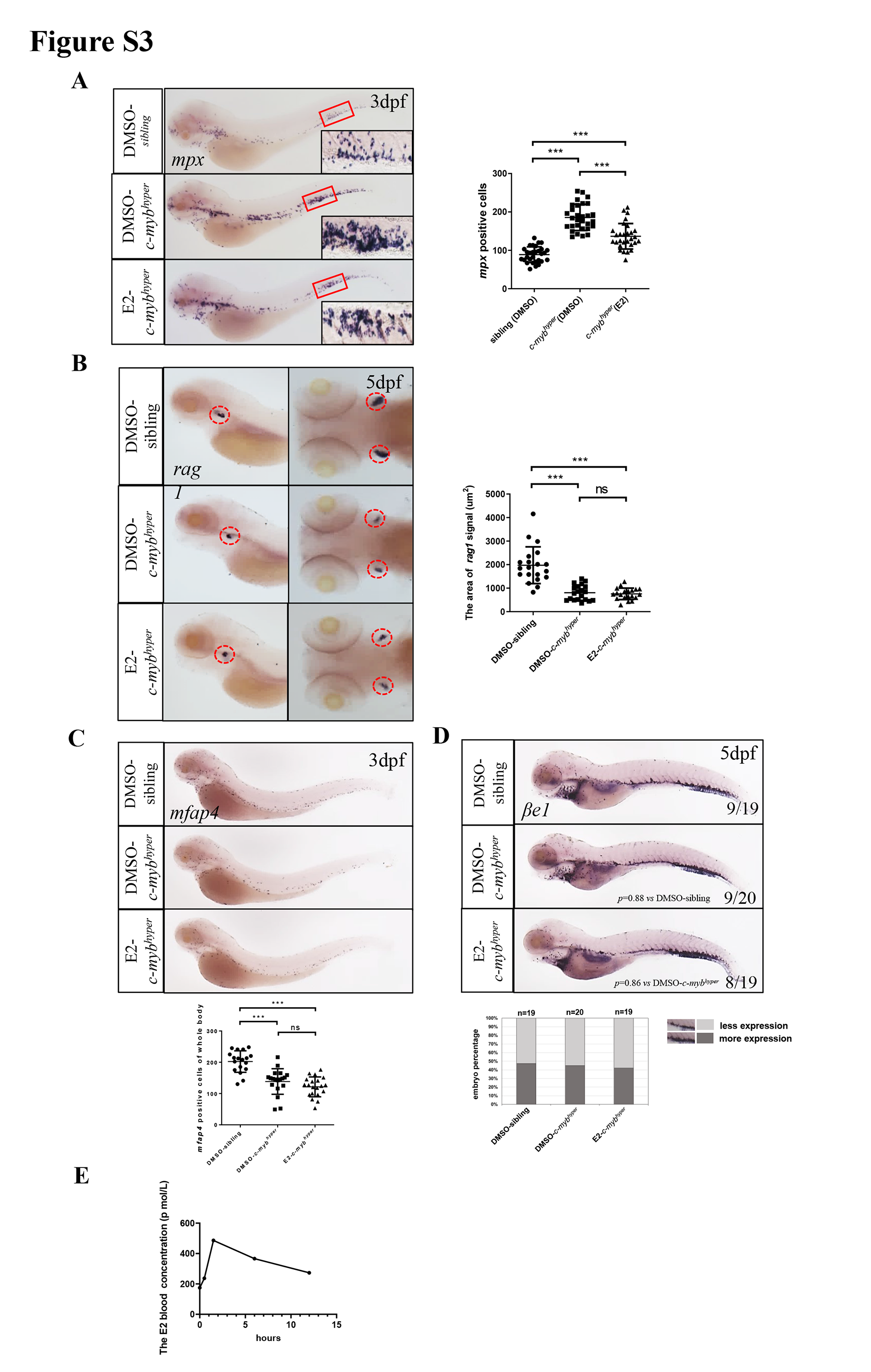
**

**
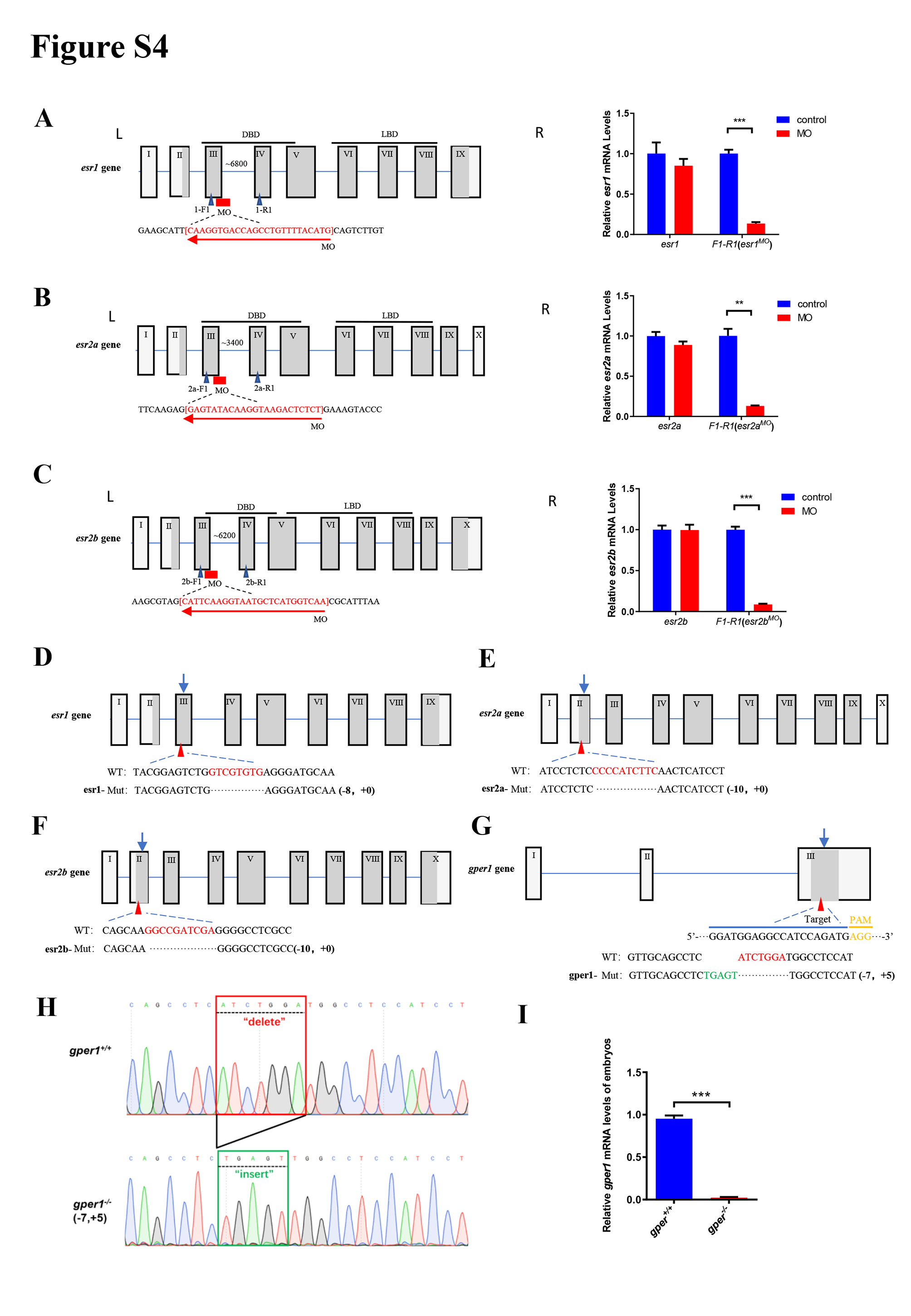

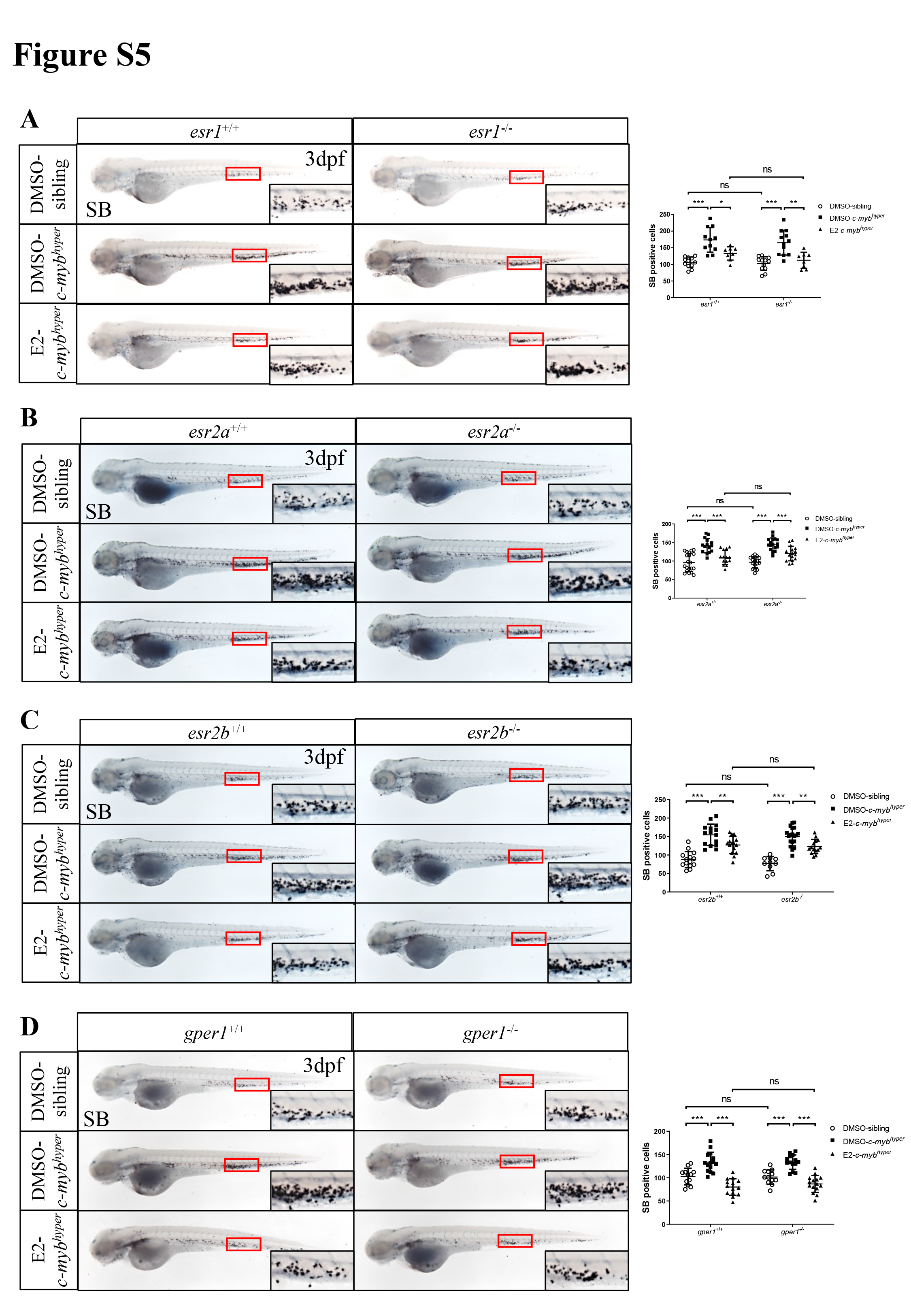
**

**
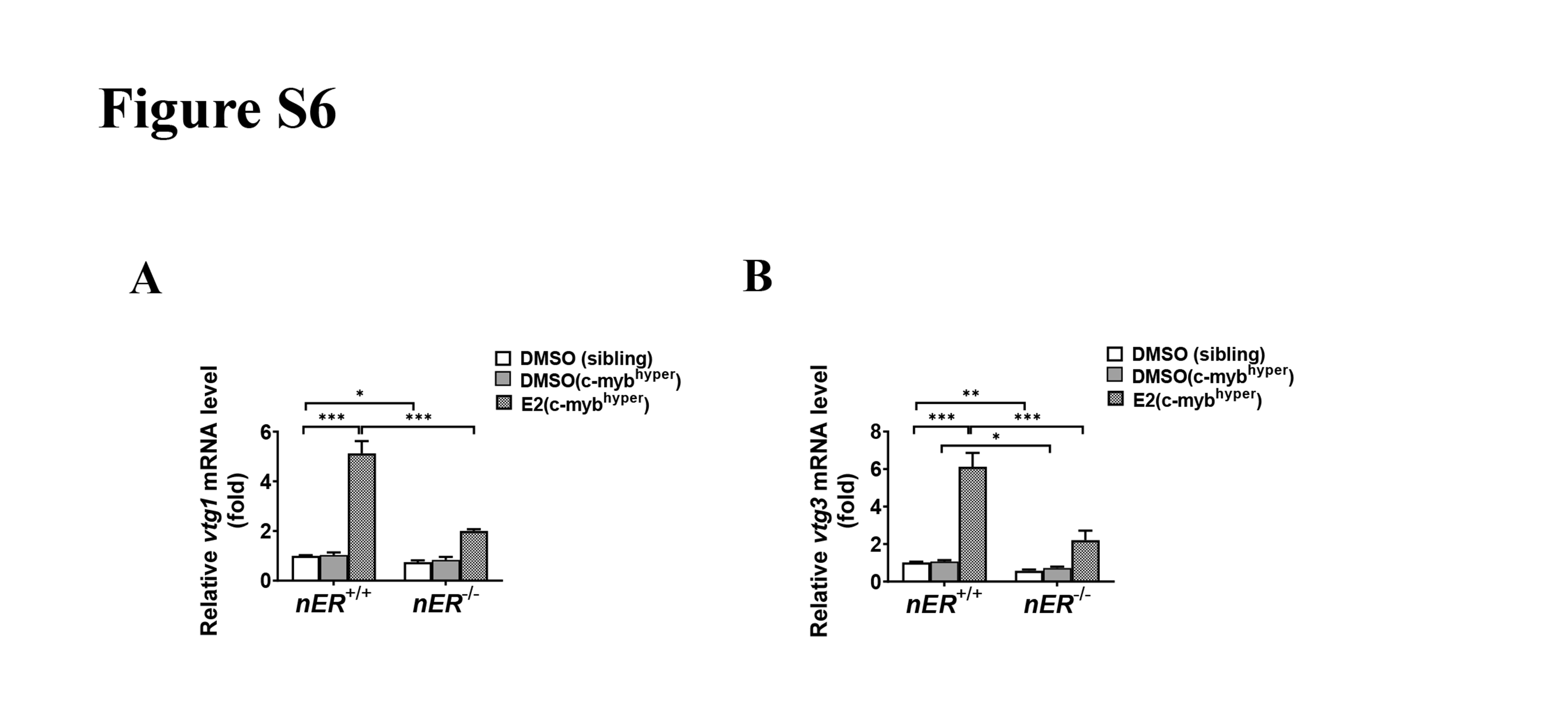
**

**
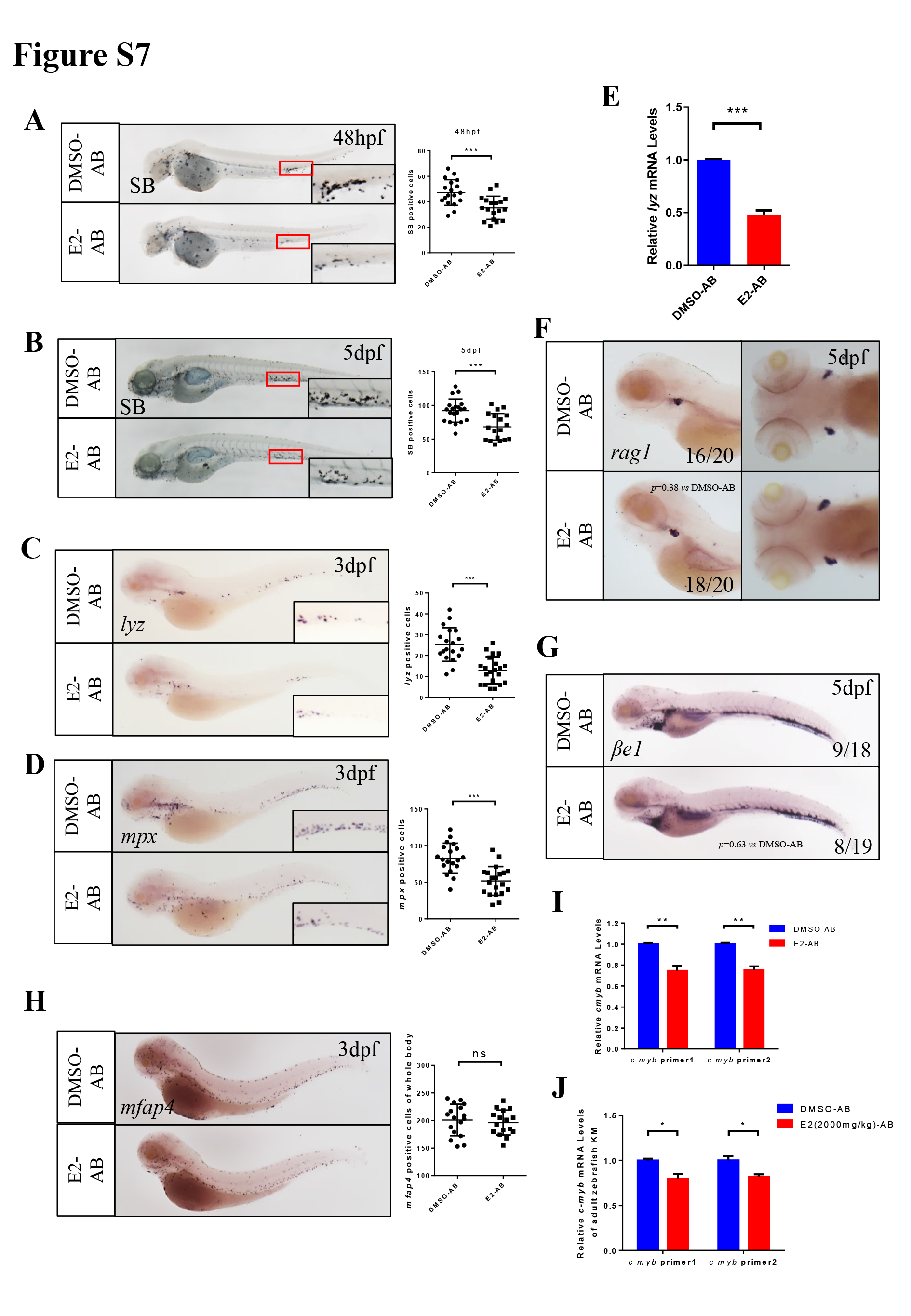
**

**Supplementary table 1：qPCR (Refers to Quantitative RT-PCR) primers and genotyping (HRM) primers**

| Gene | Forward | Reverse |
| --- | --- | --- |
| *ef1a* | GAGAAGTTCGAGAAGGAAGC | CGTAGTATTTGCTGGTCTCG |
| *c-myb*-primer1 | GCACGAGAAACTTGGAAACCGATG | GCTTGGTGTGTGATTGTAGTTC |
| *c-myb*-primer2 | AGTCCTTTGCAGCAGTTGAACAC | CGTTCATATGACCAGAGCTCGCG |
| *lyz* | AAAGCAGGTTTAAGACCCAC | CCAGGTTTCCCATGATTTCAG |
| *gper1*  *vtg1*  *vtg3* | GCAGCATCTTCTTCCTGACATG  ACTACCAACTGGCTGCTTAC  CAGATGGCTTTATCGGCGTGAC | GACATTGGCGAAGCAGAAGTG  ACCATCGGCACAGATCTTC  CACGGCAGGCCCATTGAAAC |
| *esr1* | F1:CGCCTCTGGATATCATTACGGAGT | R1:ACGGTTTCTGTCAATAGTGCACT |
| *esr2a* | F1:ACTGTGCCGTCTGCAGTGATTAC | R1:GCGGTTCTTGTCGATAGTGC |
| *esr2b* | F1:TGTGCCATGACTACGCTTCTGG | R1:GCCTGACAGCTCTTGCGTCTG |
| *esr1*(HRM) | CCCTAAGGAGGAGCACAGCG | CGAGGTGCCGAAGGTCTGTG |
| *esr2a*(HRM) | ATGTCCGAGTATCCCGAAGGAG | GTCTGTGTAGGGCGATGGGAT |
| *esr2b*(HRM) | ATGAGCTCCTCCCCTGGACCTG | GCCTCCACATATGGGGAAGGAATG |
| *gper1*(HRM) | TGCGAACCATGCAGCATGC | CCGGTATGTTGCGTCTGCAC |

**Supplementary table 2：Morpholino sequences (Refers to Morpholino injections)**

| Gene | Morpholino | Reference: |
| --- | --- | --- |
| *esr1* | 5’ CATGTAAAACAGGCTGGTCACCTTG 3’ | [1] |
| *esr2a* | 5’ AGAGAGTCTTACCTTGTATACTC 3’ | [1] |
| *esr2b* | 5’ TTGACCATGAGCATTACCTTGAATG 3’ | [1] |
| *gper1* | 5’ TCACATTGGTAGTCTGCTCCTCCAT 3’ | [2] |
| *hif1aa* | 5’ TTTTCCCAGGTGCGACTGCCTCCAT 3’ | [3] |
| *hif1ab* | 5’ACCCTACAAAAGAAAGAAGGAGAGC 3’ | [3] |

**References**

1. Griffin LB, January KE, Ho KW, Cotter KA, Callard GV. Morpholino-Mediated Knockdown of ER alpha, ER beta a, and ER beta b mRNAs in Zebrafish (Danio rerio) Embryos Reveals Differential Regulation of Estrogen-Inducible Genes. Endocrinology. 2013;154:4158-69.

2. Shi Y, Liu X, Zhu P, Li J, Sham KWY, Cheng SH, et al. G-protein-coupled estrogen receptor 1 is involved in brain development during zebrafish (Danio rerio) embryogenesis. Biochemical and Biophysical Research Communications. 2013;435:21-27.

3. Gerri C, Marass M, Rossi A, Stainier DYR. Hif-1 alpha and Hif-2 alpha regulate hemogenic endothelium and hematopoietic stem cell formation in zebrafish. Blood. 2018;131:963-73.
